# One Step In Situ Loading of CuS Nanoflowers on Anatase TiO_2_/Polyvinylidene Fluoride Fibers and Their Enhanced Photocatalytic and Self-Cleaning Performance

**DOI:** 10.1186/s11671-019-3052-5

**Published:** 2019-06-25

**Authors:** Zhi-Guang Zhang, Hui Liu, Yu-Qian Cui, Min Dong, Qing-Hao Li, Xiao-Xiong Wang, Seeram Ramakrishna, Yun-Ze Long

**Affiliations:** 1 Collaborative Innovation Center for Nanomaterials and Devices, College of Physics, Qingdao University, Qingdao, 266071 China; 2 College of Science and Information, Qingdao Agricultural University, Qingdao, 266109 China; 3 College of Environmental Science and Engineering, Qingdao University, Qingdao, 266071 China; 4 Center for Nanofibers and Nanotechnology, Faculty of Engineering, National University of Singapore, Singapore, Singapore

**Keywords:** CuS, TiO_2_, Nanoflower, Fiber, Photocatalysis

## Abstract

CuS nanoflowers were loaded on anatase TiO_2_/polyvinylidene fluoride (PVDF) fibers by hydrothermal treated electrospun tetrabutyl orthotitanate (TBOT)/PVDF fibers at low temperature. The results indicated that the amount of copper source and sulfur source determined the crystallization and morphology of the resultant products. It was found that the composite of CuS narrowed the band gap energy of TiO_2_ and enhanced the separation efficiency of the photogenerated electron-hole pairs of TiO_2_. The photocatalytic reaction rate of CuS/TiO_2_/PVDF fibers to rhodamine B was 3 times higher than that of TiO_2_/PVDF fibers under visible light irradiation. Besides, owing to the preparation process was carried out at low temperature, the flexibility of CuS/TiO_2_/PVDF fibers was ensured. In addition, the self-cleaning performance of the dye droplets on the resultant product surface was demonstrated under visible light. Meanwhile, the resultant product can automatically remove dust on the surface of the material under the rolling condition of droplets due to its hydrophobicity. Therefore, the as-prepared CuS/TiO_2_/PVDF fibers can not only degrade the contaminated compounds, but also depress the maintenance cost owing to its self-cleaning performance, which means a very practical application prospect.

**Electronic supplementary material:**

The online version of this article (10.1186/s11671-019-3052-5) contains supplementary material, which is available to authorized users.

## Introduction

In recent years, due to the unreasonable development and utilization of natural resources, the environment of human beings has been seriously polluted, including air, soil, and water pollutions. Among them, the water pollution has become one of the urgent problems to be solved because it is closely related to human life. Due to the landmark research of Fujishima and Honda [1], semiconductor photocatalysis technology has won worldwide praise as a potential solution for the degradation of toxic organic pollutants in water to achieve environmental sustainability. So far, many semiconductors have been employed in the water treatment [2–5]. Manikandan et al. synthesized MoS_2_/*α*-MoO_3_ heterostructured nanoflowers through one-step hydrothermal method [6]. The as-prepared MoS_2_/*α*-MoO_3_ heterostructured nanoflowers have a high surface area and excellent adsorbed performance. Wei and co-workers prepared porous Co_3_O_4_ nanosheets by hydrothermal method [7]. The resultant products exhibit a much lower overpotential of 318 mV at a current density of 10 mA cm^−2^. Besides, these porous Co_3_O_4_ nanosheets also display excellent electrochemical stability. Among many semiconductors, titanium dioxide (TiO_2_) has become a well-known photocatalyst because of its unparalleled efficiency and stability. However, the effectiveness of TiO_2_ is hindered by its wide band gap (Eg ~ 3.2 eV), which makes it impossible to directly utilize visible light, leading to the absence of TiO_2_ as a visible photocatalyst. In addition, the rapid recombination of photogenerated charge carriers in TiO_2_ greatly reduces its light quantum efficiency.

In order to alleviate these problems, a lot of researches have been carried out to enhance the visible light photocatalytic ability of TiO_2_ [8–13]. The combination of TiO_2_ with other narrow-gap semiconductor components, particularly, two dimensional (2D) narrow-gap semiconductor components, has been proved to be a flourishing strategy in improving the separation efficiency of photoinduced charge carriers [14–21]. Ouyang et al. synthesized BiOCl nanosheets/TiO_2_ nanotube arrays heterojunction by the combination of anodization process and impregnation method [22]. The as-prepared products enhanced the separation efficiency of photogenerated charge carriers through the interface between the BiOCl nanosheets and TiO_2_ nanotube arrays. In addition, the combination of 2D graphene with good conductivity and TiO_2_ can also obtain good photocatalytic performance [23]. Interestingly, research on the complexation with metal sulfides has made some progress [24–29]. Particularly, the TiO_2_ samples composited cadmium and lead semiconductor have achieved remarkable performance, but also caused serious secondary pollution problems. Therefore, the exploration of benign narrow band gap semiconductor composites is imperative.

Copper sulfide (CuS), a semiconductor material with a band gap of 2.0 eV, has excellent performance and has been used in solar cells, photocatalysis, lithium batteries, etc. [30, 31]. Therefore, the coupling of CuS and TiO_2_ to prepare a CuS-TiO_2_ composite with visible light activity and high separation efficiency of photogenerated carriers provides a possibility. Yu and co-workers prepared two different composites TiO_2_-CuS-a and TiO_2_-CuS-b through direct deposition and bifunctional linker coupling methods on TiO_2_ nanospheres [32]. Compared with TiO_2_-CuS-a prepared through direct deposition method, TiO_2_-CuS-b with a regular “spiky-ball-like” structure has enhanced disinfection ability, which improved its photocatalytic performance under solar and UV light. Lu et al. prepared CuS nanoflowers loaded on rutile TiO_2_ using copper and sulfur powder through element-direct-reaction [33]. Compared with pure TiO_2_ or copper sulfide, the as-synthesized CuS/TiO_2_ samples presented enhanced photocatalytic performance due to the formation of hetero-junction between CuS and rutile TiO_2_. Hou et al. prepared TiO_2_ fibers by electrospinning and post-sintering. The prepared TiO_2_ fibers were soaked in NaOH solution and then grew CuS particles on the surface of the fibers by hydrothermal method [34]. The island-like CuS particles attached to TiO_2_ nanofibers which had a diameter less than 100 nm to form heterostructures. The as-prepared samples showed improved photocatalytic activity for the degradation of the methyl blue (MB) dye. The above-mentioned CuS-TiO_2_ composites are powdered materials or very brittle fiber materials after high-temperature treatment. These materials are difficult to separate and recycle after photocatalytic experiment in water. In addition, photocatalytic materials often suffer from contamination by target pollutants, resulting in a decrease in their photocatalytic performance.

In this work, CuS nanoflowers were loaded on the TiO_2_/polyvinylidene fluoride (PVDF) fibers by one-step hydrothermal treatment on electrospun tetrabutyl orthotitanate (TBOT)/PVDF fibers at low temperature. On the one hand, the preparation process is convenient and simple. On the other hand, the low temperature use in the preparation process guarantees the flexibility of PVDF. The as-prepared CuS/TiO_2_/PVDF fiber has good visible light photocatalytic performance. Under visible light, the photocatalytic reaction rate of CuS/TiO_2_/PVDF fibers to rhodamine B (RhB) is 3 times higher than that of TiO_2_/PVDF fibers. In addition, the self-cleaning performance of the resultant product was investigated. It can be concluded that the as-prepared CuS/TiO_2_/PVDF fibers have good separability, recyclability, and self-cleaning performance. The resultant samples in this work provide a new perspective for exploring the application of novel flexible, recyclable, and self-cleaning photocatalytic materials for environmental pollution controllability.

## Methods/Experimental

### Materials

PVDF (FR904) was purchased from Shanghai 3F New Materials Co., Ltd., and N, N-dimethylformamide (DMF, AR, 99.5%), acetone (CP, 99.0%), TBOT (CP, 98.0%), copper nitrate (Cu (NO_3_)_2_·3H_2_O, AR, 99.0%), thiourea (AR, 99.0%), RhB, MB, and methyl orange (MO) were purchased from Sinopharm Chemical Reagent Co., Ltd. All reagents were used as received without any further purification.

### Preparation of TBOT/PVDF Fibers

In a typical electrospinning process, 4.0 g PVDF powder was mixed with 10 g acetone and 10 g DMF. Then, the mixture was stirred vigorously at 40 °C until it became clear and transparent. After that, 10 ml of TBOT was added to the solution mentioned above and stirred for 1 h at 40 °C to form the TBOT/PVDF precursor solution. A 5.0-ml syringe containing TBOT/PVDF precursor solution with a blunt metal needle was placed on a propeller. The propulsion speed was set at 1.8 ml h^−1^. The fiber collector was a stainless steel roll wrapped in a piece of aluminum foil with a rotate speed of about 250 rpm. A DC voltage source set to 9 kV was placed between the tip and the collector at a distance of 11 cm. The as-prepared TBOT/PVDF fibers were dried at 60 °C for 10 h to eliminate any remaining solvent. Finally, the as-prepared TBOT/PVDF fibers were cut into 2.5 cm × 2.5 cm pieces for subsequent hydrothermal treatment.

### Fabrication of CuS/TiO_2_/PVDF Fibers

Cu (NO_3_)_2_·3H_2_O and thiourea were added into 30 ml of deionized water in a fixed molar ratio (1:2) and stirring was continued for 30 min. Then, the solution was transferred to the 50-ml stainless steel autoclave, and the cut TBOT/PVDF pieces were placed inside. The stainless steel autoclave was placed in an electric oven and heated at 150 °C for 24 h. In the hydrothermal process, on the one hand, TBOT in TBOT/PVDF is hydrolyzed to form TiO_2_/PVDF. On the other hand, CuS is continuously growing on the surface of TiO_2_/PVDF to form CuS/TiO_2_/PVDF. Finally, the as-prepared fibers were thoroughly washed with ethanol and deionized water, then dried in an electric oven at 60 °C for 10 h and the flexible CuS/TiO_2_/PVDF fibers were obtained (shown in Additional file 1: Figure S1. For comparison, the amount of Cu (NO_3_)_2_·3H_2_O added was 0.1, 0.5, and 1 mmol, respectively. Correspondingly, the as-synthesized materials were named as Cu 0.1, Cu 0.5, and Cu 1, respectively.

### Characterization

The X-ray diffraction (XRD) patterns were carried out on a Rigaku SmartLab X-ray diffractometer in the 2θ range of 10–90° using Cu-Kα radiation (*λ* = 1.54178 Å) at an accelerating voltage of 40 kV. In addition, the scanning electron microscopy (SEM) images and transmission electron microscopy (TEM) images of the as-prepared samples were obtained from Phenom Pro scanning electron microscope and JEOL JEM-2100 Plus transmission electron microscope, respectively. To achieve a detailed understanding of the chemical composition information of the as-prepared products, the X-ray photoelectron spectroscopy (XPS) detections were operated on a Thermo Scientific Escalab 250Xi system. The light-harvesting performance of the as-prepared samples was evaluated by diffuse reflectance spectra (DRS). DRS investigations were carried out on Shimadzu UV-2600 spectrophotometer equipped with an integrating sphere accessory and using BaSO_4_ as a diffuse reflectance standard. The photoluminescence spectra (PL) of the resultant samples were studied on a Hitachi F-2500 fluorescence spectrometer with a Xe lamp at room temperature using an excitation wavelength of 320 nm.

### Photocatalytic Activity

The photocatalytic performances of the resultant products were investigated under a 9-W white light LED irradiation at room temperature by degrading RhB (5 mg L^−1^) which was directly prepared with RhB reagent and deionized water. The light spectrum of this LED is shown in Additional file 1: Figure S2. In order to show the effect of the amount of Cu sources on photocatalysis, the film photocatalysts with the same area were used in photocatalysis experiments. In photodegradation experiments, a piece of the resultant product was put into a 100-ml quartz tube with 60 ml RhB solution followed by 30-min magnetic stirring in the dark to ensure the adsorption-desorption equilibrium between RhB and the resultant product. Then, the quartz tube was placed under the light source with a distance of 4.0 cm. At given time intervals, 3 ml solution was taken out from the quartz tube followed by centrifuging to remove the particles. Then, the concentration of RhB remained in the solution was investigated its absorbance at 554 nm on a Shimadzu UV-2600 spectrophotometer. In order to ensure a roughly equivalent volume of solution, the analyzed solution was quickly poured back into the quartz tube after every assay.

The ratio of RhB concentration *C* at each interval to the initial concentration *C*_0_ was used to indicate the photocatalytic degradation efficiency, which was expressed as *C*/*C*_0_. After the photocatalytic degradation experiment, the resultant product was washed with ethyl alcohol and deionized water followed by drying in air for the next photodegradation process to study the recycle stability performance of the resultant product.

### Self-Cleaning Performance

#### Wetting Property

The wetting property of CuS/TiO_2_/PVDF fibers is obtained by testing the contact angle of the droplets (including H_2_O, RhB, MO, and MB) on the product under ambient temperature through a Theta Attension optical contact angle instrument.

#### Self-Cleaning Performance

Self-cleaning performance of the resultant product is evaluated by degrading surface dye droplets and removing surface dust. The RhB, MO, and MB dye droplets with a concentration of 10 mg L^−1^were dripped onto the CuS/TiO_2_/PVDF fibers and irradiated under the 9-W white lights LED. At given time intervals, an optical photograph was taken, which was used to study the self-cleaning performance of the as-prepared product to the surface contamination. For the surface dust removal of the CuS/TiO_2_/PVDF fibers, the dust was scattered on the surface of the sample before measurement. Then, a drop of water was dropped on the surface of the product. The sample was tilted slightly to make the droplet move on the sample surface and take away the dust, thus making the material surface clean.

## Results and Discussion

### Structure and Morphology Characteristics

**Fig. 1:**
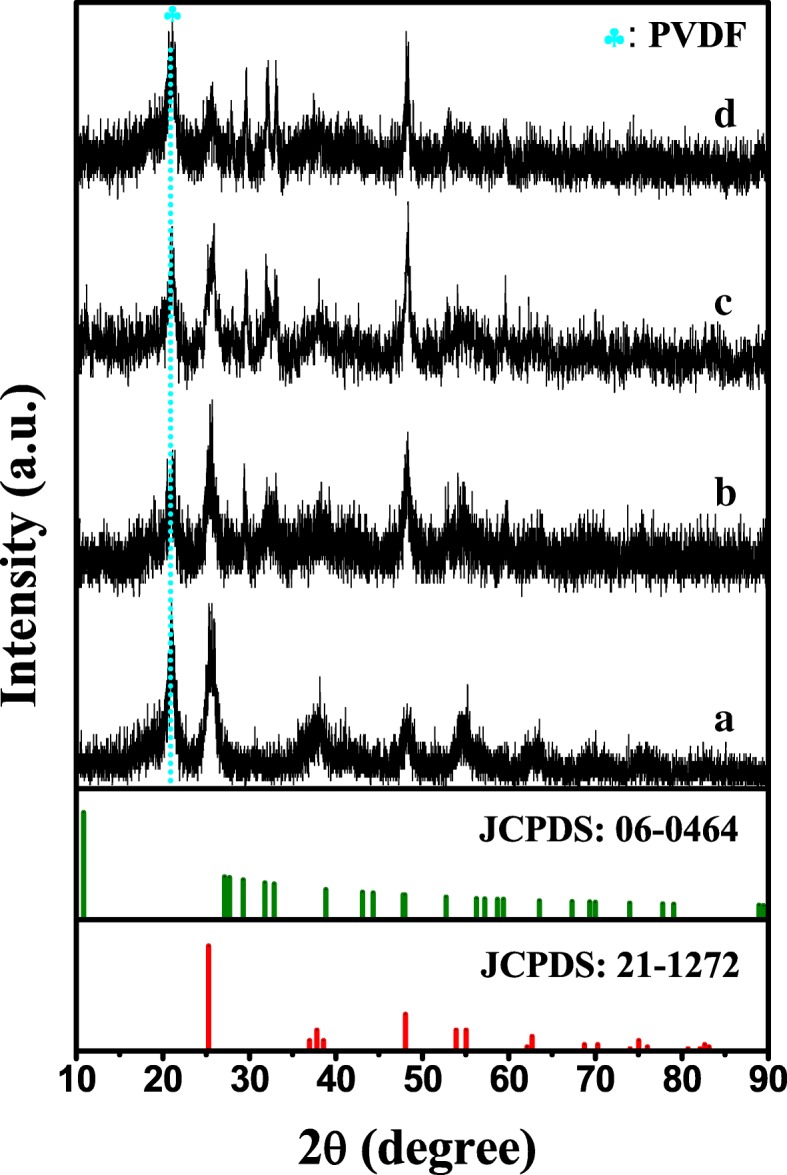
XRD patterns of (a) TiO_2_/PVDF fibers, (b) Cu 0.1, (c) Cu 0.5, and (d) Cu 1

The XRD patterns of TiO_2_/PVDF fibers, Cu 0.1, Cu 0.5, and Cu 1 are depicted in Fig. 1. It is evident that no obvious diffraction peak is detected except the diffraction peak of PVDF and TiO_2_ as displayed in Fig. 1 curve a. The diffraction peak around 20.8° can be assigned to the β phase of PVDF, and the diffraction peak at 25.67°, 37.8°, 48.2°, and 54.6° can be assigned to the (101), (004), (200), and (211) crystal faces of anatase phase TiO_2_ (PDF card 21-1272, JCPDS), respectively [35–37]. Comparing curve b with curve a, there are two obvious diffraction peaks near the diffraction angles of 29.4° and 32.6° in curve b, which can be indexed to the (102) and (006) crystal faces of hexagonal CuS (PDF card 06-0464, JCPDS). In addition, it is worth noting that the diffraction peak near 48.3 in curve b is stronger than that in curve a, which mainly due to the fact that the diffraction peaks of TiO_2_ and CuS are superimposed at this diffraction angle. These indicate that the CuS is successfully formed on the TiO_2_/PVDF fibers. Furthermore, a diffraction peak appears gradually near the diffraction angle 27.7° in curve c and d compared with curve b, which corresponds to (101) crystal faces of CuS. Meanwhile, the diffraction peak near 32.6° also gradually become two distinct peaks at 31.9° and 32.9°, respectively, corresponding to (103) and (006) crystal faces of CuS. Based on the results mentioned above, it can be found that with the increase of Cu and S sources, the crystallization of CuS becomes better.

**Fig. 2:**
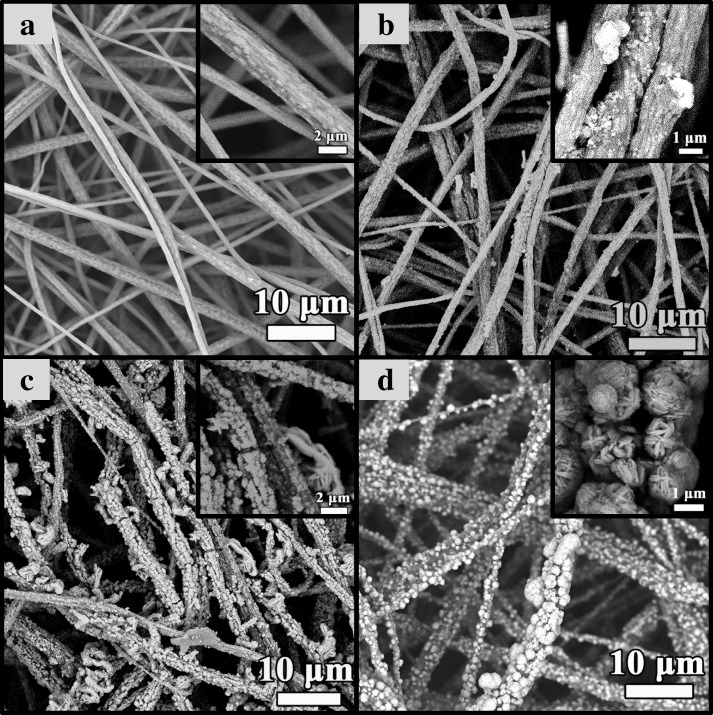
SEM images of **a** TiO_2_/PVDF fibers, **b** Cu 0.1, **c** Cu 0.5, and **d** Cu 1

The typical SEM images of TiO_2_/PVDF fibers, Cu 0.1, Cu 0.5, and Cu 1 are displayed in Fig. 2. As can be seen from Fig. 2a, the TiO_2_/PVDF fibers are arranged in disorder with a relatively rough surface (displayed in the inset) which mainly due to the formation of TiO_2_ in the fibers. Comparing Fig. 2b with Fig. 2a, the surface of fibers in Fig. 2b becomes rougher, and a small amount of CuS particles have appeared on the surface of fibers which can be easily found in the inset illustration. When the amount of Cu source increases to 0.5 mmol, a very large change can be found in comparison with Fig. 2b that a large amount of CuS particles coat on the surface of TiO_2_/PVDF fibers, as shown in Fig. 2c. Furthermore, it can be seen from the inset illustration that a small amount of hexagonal layered CuS nanoflowers appears on the surface of TiO_2_/PVDF fibers. When the amount of Cu source increase to 1 mmol, obviously, a large number of aggregated CuS particles have appeared on the fiber surface (depicted in Fig. 2d). Through careful examination of the inset of Fig. 2d, these large CuS nanoflowers are composed of many hexagonal lamellar CuS. Based on the above results, it can be known that the increase of Cu source, on the one hand, continuously increases the amount of CuS on the surface of the fiber and, on the other hand, enhances the crystallization and growth of CuS on the surface of the fiber leading to the morphology of CuS gradual change from particles to hexagonal lamellar structure.

**Fig. 3:**
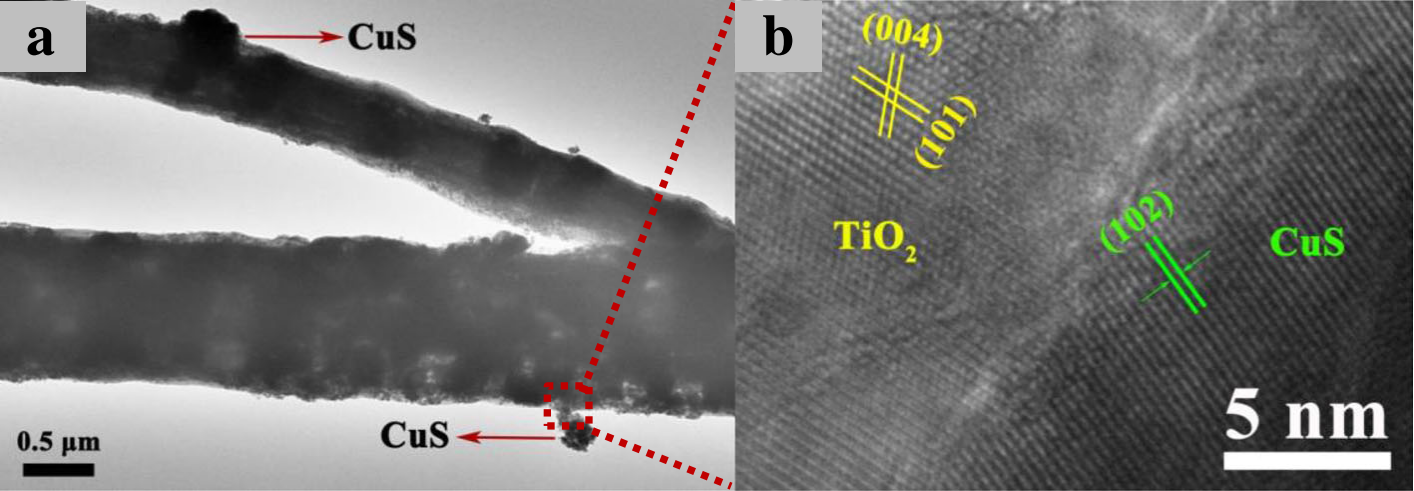
TEM image of Cu 1 **a** and high-resolution TEM image of Cu 1 **b**

The morphology of CuS/TiO_2_/PVDF fiber is further investigated by TEM and HRTEM, as presented in Fig. 3. As depicted in Fig. 3a, it is evident that some CuS particles with uneven size are distributed on the surface of TiO_2_/PVDF fiber. The contact position between CuS and TiO_2_/PVDF fiber is enlarged to obtain a high-resolution image, as shown in Fig. 3b. From the high-resolution TEM image, it can be found that CuS and TiO_2_ have obvious crystal boundary. By measuring the interplanar spacing, it can be found that there are mainly two kinds of crystal faces of TiO_2_ with the interplanar spacing of 0.35 and 0.23 nm, respectively, corresponding to the (101) and (004) crystal faces of TiO_2_. The angle between these two crystal faces is about 68°, which is consistent with the literature [38]. In addition, there is mainly a crystal face with a crystal interplanar spacing of about 0.31 nm, corresponding to the (102) crystal face of CuS. Therefore, the TEM and HRTEM studies are consistent with the XRD measurements.

**Fig. 4:**
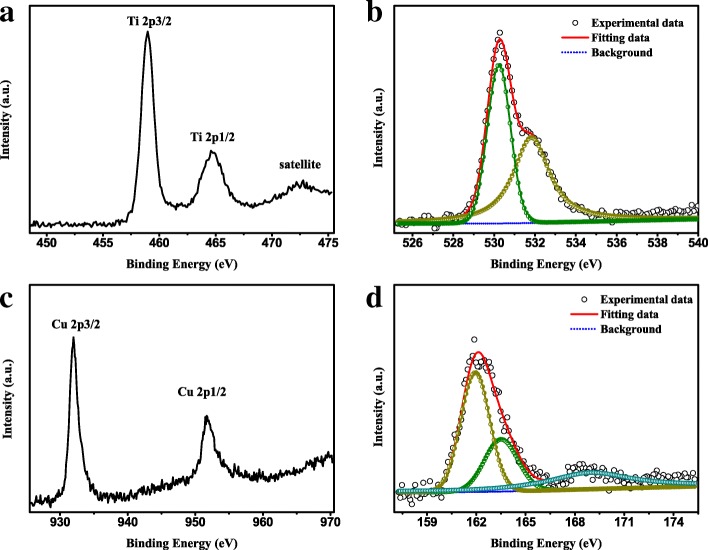
High-resolution XPS spectra of **a** Ti 2p, **b** O 1s, **c** Cu 2p, and **d** S 2p in Cu 1 sample

XPS analysis is employed to study the chemical composition information and the bonding configuration of the resultant products. Characteristic peaks of Cu, S, Ti, O, F, and C can be clearly detected from the XPS survey spectra, as shown in Additional file 1: Figure S3. The high-resolution XPS spectra of Ti 2p, O 1s, Cu 2p, and S 2p are illustrated in Fig. 4. As can be seen in Fig. 4a, the peaks located at 459.0 and 464.7 eV can be assigned to Ti 2p_3/2_ and Ti 2p_1/2_, respectively [39]. The peak of O1s can be divided into two peaks (displayed in Fig. 4b), respectively, corresponding to Ti-O of TiO_2_ (530.2 eV) and hydroxyl group (531.8 eV) in the as-prepared product [40, 41]. In Cu 2p profiles (Fig. 4c), the peaks located at 932.0 and 952.0 eV are indexed to 2p_3/2_ and 2p_1/2_ of Cu^2+^, respectively [42]. Meanwhile, for the peaks of S element (depicted in Fig. 4d), the broad spectra located around 162.1 eV can be broken into two peaks of 161.9 and 163.5 eV, respectively, corresponding to the 2p_3/2_ and 2p_1/2_ of S^2−^ [43]. In addition, a weak peak at 168.8 eV is detected which may be the intermediate products produced by thiourea in hydrothermal reaction [44]. Furthermore, the atomic concentration from high-resolution XPS spectra is depicted in Table 1. It is easy to find that the ratio of O to Ti atoms is more than 2:1, which is mainly due to the presence of O atoms in PVDF [45]. The ratio of S to Cu atoms is about 1.27, which is a little more than 1:1. The main reason is that the excessive S source (the ratio of Cu source to S source is 1:2) is used in the preparation process, resulting in some S remaining on the surface of the sample. The XPS results mentioned above further confirm the presence of CuS, TiO_2_, and PVDF in the Cu 1 sample, which agree well with the XRD and TEM results.

**Table 1:** The atomic concentration of element in Cu 1 sample

Element	C	F	Ti	O	Cu	S
Atomic concentration	50.08	28.81	4.01	11.58	2.43	3.09

### Optical Characteristics

**Fig. 5:**
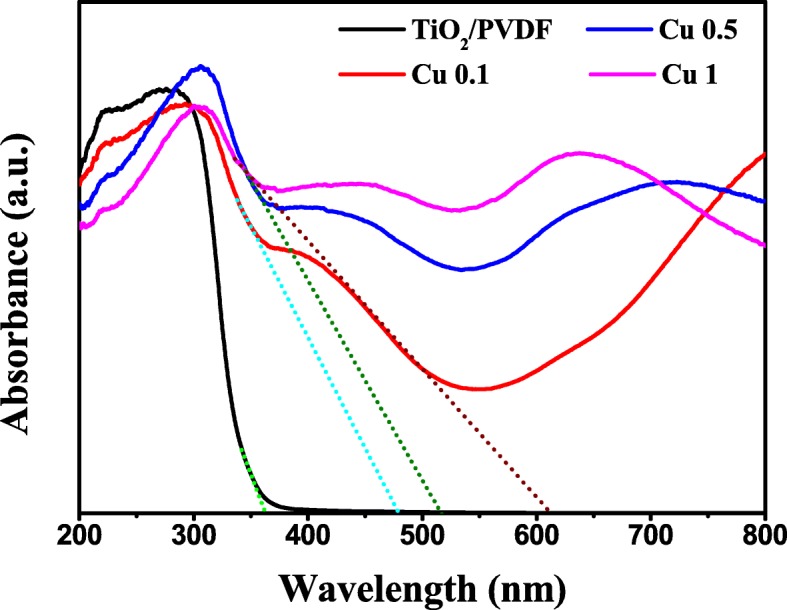
UV-Vis diffuses reflectance spectra of TiO_2_/PVDF, Cu 0.1, Cu 0.5, and Cu 1

The optical characteristics of TiO_2_/PVDF, Cu 0.1, Cu 0.5, and Cu 1 are studied by UV-Vis diffuse reflectance spectra which are transformed to absorbance values using the Kubelka-Munk function [35, 46], as illustrated in Fig. 5. It is easy to find that the absorbance value of the samples composited CuS is much higher than that of TiO_2_/PVDF fibers in the visible light region, implying that the composite of CuS greatly enhances the light harvest performance of TiO_2_. Furthermore, the absorbance value of samples composited CuS increases continuously with the increase of the amount of Cu source.

According to the previous literature, anatase TiO_2_ belongs to indirect band gap semiconductor, while CuS belongs to direct band gap semiconductor [47, 48]. Their energy band gap (Eg) is measured by equation Eg = 1240/*λ*_g_ (eV), where *λ*_g_ is the absorption edge calculated from the intercept between the tangent of the absorption curve and the abscissa coordinate [49]. The absorption edge and energy band gap of the resultant samples are depicted in Table 2.

**Table 2:** Absorption edge and energy band gap of TiO_2_/PVDF, Cu 0.1, Cu 0.5, and Cu 1 samples

Typical sample	Absorption edge (nm)	Energy band gap (eV)
TiO_2_/PVDF	363	3.4
Cu 0.1	479	2.6
Cu 0.5	517	2.4
Cu 1	612	2.0

As can be seen from Table 2, the energy band gap of CuS/TiO_2_/PVDF fiber is smaller than that in TiO_2_/PVDF fiber, and the absorption edge of CuS/TiO_2_/PVDF fiber shifts to the long wavelength gradually with the increase in the amount of Cu source. It is possible that with the increase in the amount of Cu source, CuS is more and more tightly wrapped on the surface of TiO_2_/PVDF fiber (as depicted in SEM images), which makes the interface contact between TiO_2_ and CuS become larger, resulting in the absorption edge of TiO_2_ moving to a long wavelength [50]. Besides, with the increase of Cu source, the crystalline of CuS becomes better which reduces the energy band gap of the resultant product [51, 52].

**Fig. 6:**
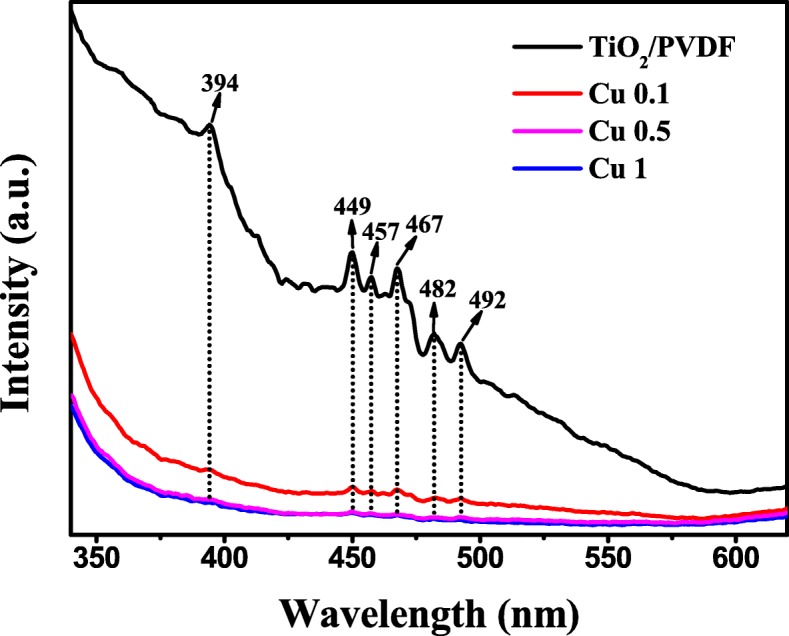
PL spectra of TiO_2_/PVDF, Cu 0.1, Cu 0.5, and Cu 1

The PL spectra are usually employed to investigate the recombination of photo-generated electron-hole pairs in the semiconductor [53, 54]. The PL spectra of TiO_2_/PVDF, Cu 0.1, Cu 0.5, and Cu 1 samples are displayed in Fig. 6. It can be found in Fig. 6 that there is an emission peak around 394 nm (~ 3.2 eV) in the TiO_2_/PVDF fiber and Cu 0.1 also has a weak emission peak here. The position of this emission peak is close to the band-edge emission of TiO_2_ in these two samples and should be the near-band-edge emission of TiO_2_ [55, 56]. In Cu 0.5 and Cu 1 samples, these emission peaks disappear. In addition, there are five emission peaks in the TiO_2_/PVDF fiber between 449 nm (2.76 eV) and 492 nm (2.52 eV), which should be caused by oxygen vacancy (V_O_) defects generated during the preparation process [57, 58]. In the samples of Cu 0.1, Cu 0.5, and Cu 1, these five emission peaks still exist, while the intensity of these five emissions sharply declines, mainly due to the CuS covers on the surface of the TiO_2_/PVDF fiber and weakens the intensity of the emission peak.

Besides, the intensity of the PL spectra of the CuS/TiO_2_/PVDF fibers is much lower than that of the TiO_2_/PVDF fiber, proving that the composite of CuS increases the separation efficiency of photogenerated electron-hole pairs and suppresses the recombination of photogenerated electron-hole pairs, which also means that photocatalytic ability of TiO_2_/PVDF fiber is enhanced.

### Photocatalytic Performances

**Fig. 7:**
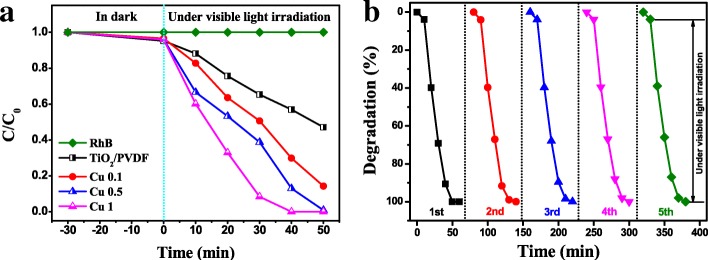
**a** Photocatalytic degradation curves of RhB over the samples: RhB without photocatalyst, TiO_2_/PVDF, Cu 0.1, Cu 0.5, and Cu 1. **b** The recycle experiment using the sample Cu 1

The photocatalytic performances of the as-prepared samples are depicted in Fig. 7. It can be seen in Fig. 7a that the concentration of RhB does not change under visible light, which meant that it was very stable under visible light without spontaneous degradation. However, it is interesting to note that TiO_2_/PVDF fiber should have only ultraviolet photocatalytic ability but has visible light photocatalytic ability, and its photodegradation of RhB reached 52.9% in 50 min. Compared with TiO_2_/PVDF fiber, the Cu 0.1 and Cu 0.5 samples have a faster photocatalytic rate, and the photocatalytic efficiencies of RhB in 50 min are 85.7% and 99.2%, respectively. As can be seen from Fig. 7a, the Cu 1 sample has the highest photodegradation rate, which degrades RhB completely in 40 min.

In addition, the photocatalytic degradation of RhB can also be represented by a pseudo-first-order kinetics process. The equation is as follows [59]:

$$ -\ln \left(C/{C}_0\right)= kt $$

where *k* and *t* are the reaction rate constant and measurement time, respectively; *C*_0_ and *C* are the concentration of RhB at initial and *t* time. According to the equation, the reaction rate constant of RhB degradation by the as-prepared photocatalyst is calculated, as shown in Additional file 1: Figure S4. It can be seen that the reaction rate constant of Cu1, Cu0.5, Cu0.1, and TiO_2_/PVDF is 2.9 × 10^−2^, 1.8 × 10^−2^, 1.6 × 10^−2^, and 9.8 × 10^−3^ min^−1^, respectively. It is obvious that the reaction rate of Cu1 is about 3 times higher than that of TiO_2_/PVDF.

The photocatalytic recycle experiments are carried out on the Cu1 sample for 5 times, as displayed in Fig. 7b. It can be seen that the photocatalytic efficiency of Cu1 sample decreases slightly at 40 min, but remains 100% at 50 min, implying the as-prepared product has a certain recycled stability.

Usually, the photo-induced holes (h^+^), superoxide anion radicals (${\mathrm{O}}_2^{-\cdotp }$), and hydroxyl radicals (OH·) are considered as the main active species in the photocatalytic process. [60] Here, in order to investigate the photocatalytic mechanism of the TiO_2_/PVDF and CuS/TiO_2_/PVDF fibers, control experiments are carried out using ethylenediaminetetraacetic acid (EDTA), nitrogen (N_2_), and tertiary butanol (tBuOH) as the individual scavengers for h^+^, ${\mathrm{O}}_2^{-\cdotp }$, and OH·, respectively, as displayed in Additional file 1: Figure S5. It can be seen that ${\mathrm{O}}_2^{-\cdotp }$ have the greatest influence on photocatalytic efficiency in the TiO_2_/PVDF system, followed by h^+^ and OH·. Whereas in the CuS/TiO_2_/PVDF system, h^+^ have the greatest influence on photocatalytic efficiency followed by OH· and ${\mathrm{O}}_2^{-\cdotp }$.

**Fig. 8:**
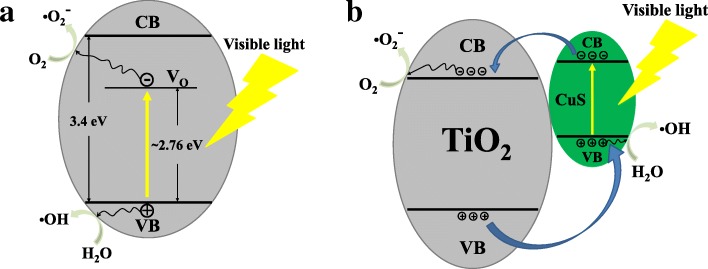
Diagram of the separation and transmission process of photogenerated electron-hole in **a** TiO_2_/PVDF fiber and **b** CuS/TiO_2_/PVDF fiber

A possible mechanism is proposed to be responsible for the photocatalytic mechanisms of the TiO_2_/PVDF fiber and CuS/TiO_2_/PVDF fiber under visible light irradiation as depicted in Fig. 8. For the TiO_2_/PVDF fiber, there are a large number of oxygen vacancy defects (V_O_) in TiO_2_/PVDF fiber, which can be confirmed in the PL spectra. These oxygen vacancy defect levels lie between the valence band (VB) and conduction band (CB) of TiO_2_. When irradiated under visible light, the electrons are excited from the VB of TiO_2_ and trapped by these defects, leaving a hole in the VB of TiO_2_. A part of the photogenerated electrons and holes migrate to the surface of TiO_2_ and react with oxygen molecules in the solution to form ${\mathrm{O}}_2^{-\cdotp }$ or react with water molecules to form OH·, thereby degrading RhB.

Since the narrow band gap CuS is coated on the surface of the TiO_2_/PVDF fiber, the CuS absorbs visible light so that electrons on the VB of CuS are excited to its CB, leaving holes in the VB. Owing to the CB energy of TiO_2_ is lower than that of CuS, electrons will be transferred from the CB of CuS to the CB of TiO_2_, and holes will be transferred from the VB of TiO_2_ to the VB of CuS [40]. The electrons transferred to the CB of TiO_2_ react with the oxygen molecules in the solution to form ${\mathrm{O}}_2^{-\cdotp }$. And the holes that migrate to the VB of CuS react with the water molecules in the solution to form OH·, thereby degrading RhB.

The composite CuS to the TiO_2_/PVDF fiber narrows the band gap of the material and enlarges the absorption range of light on the one hand. On the other hand, the ability of the material to separate photogenerated electron holes is improved, implying the photocatalytic ability is enhanced.

### Self-Cleaning Performances

**Fig. 9:**
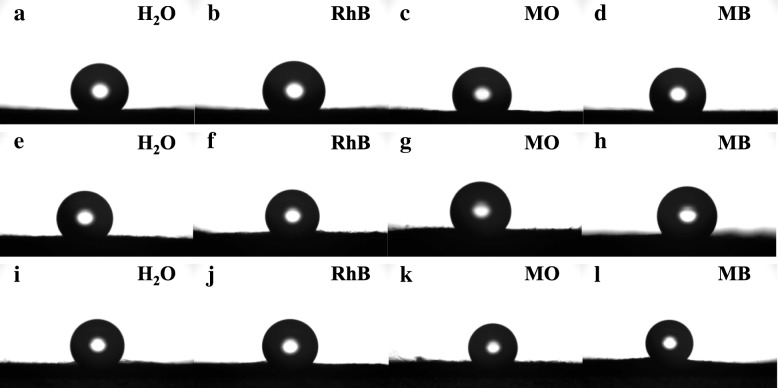
The optical images of static contact angle for **a**–**d** TiO_2_/PVDF fibers, **e**–**h** Cu 0.1, and **i**–**l** Cu 1

The wetting property of the surface decides the self-cleaning mechanism. The static contact angle, which is the contact angle between solid and liquid, is a main parameter to study the surface wetting behavior. The contact angles of H_2_O, RhB, MO, and MB for TiO_2_/PVDF fibers, Cu 0.1, and Cu 1 are shown in Fig. 9 and Table 3. It can be found that the three samples all show hydrophobicity. However, compared with TiO_2_/PVDF fibers, the hydrophobicity of Cu 0.1 and Cu 1 decreased slightly.

**Table 3:** The static contact angle of TiO_2_/PVDF fibers, Cu 0.1, and Cu1

Typical sample	H_2_O	RhB	MO	MB
TiO_2_/PVDF	135.63 ± 0.16	125.89 ± 0.86	126.30 ± 0.91	126.84 ± 0.18
Cu 0.1	127.10 ± 0.78	125.98 ± 0.91	124.74 ± 0.84	123.70 ± 0.69
Cu 1	125.68 ± 0.12	125.90 ± 0.48	124.25 ± 0.45	125.13 ± 0.51

**Fig. 10:**
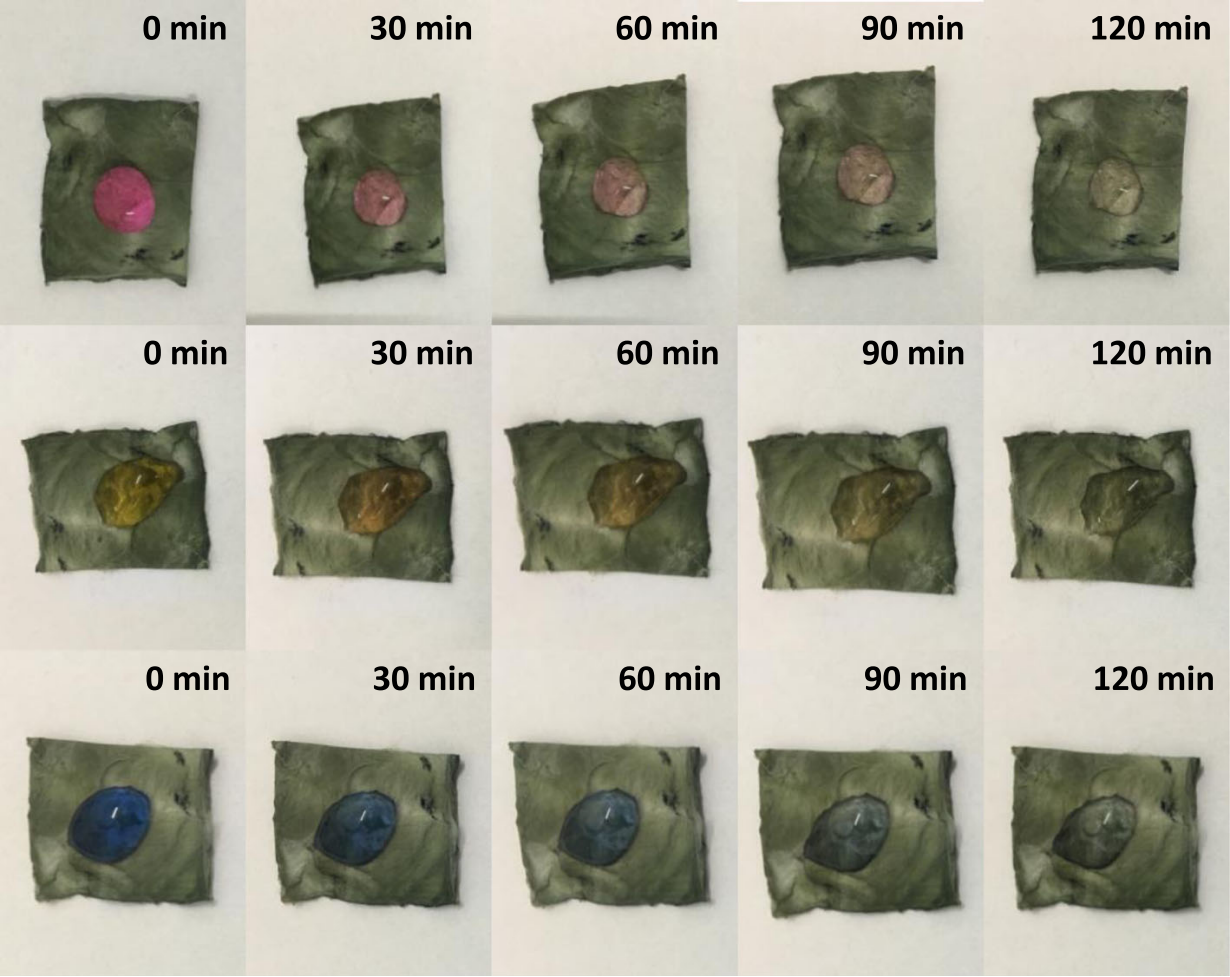
The optical photograph of the RhB, MO, and MB droplet on the surface of Cu 1 under visible light illumination

In addition, the self-cleaning properties of Cu 1 were investigated by dropping 10 mg L^−1^ of RhB, MO, and MB onto the surface of the resulted product under visible light illumination, as presented in Fig. 10. It is obviously that the colors of these dyes almost disappear in about 120 min, leaving only transparent droplets on the surface of the material. As we know, in the photocatalysis process, the photocatalysts are easily attached to dye molecules causing self-pollution, which reduces its photocatalytic performance. The as-prepared products can greatly improve the photocatalytic effect in practical use due to their self-cleaning ability.

**Fig. 11:**
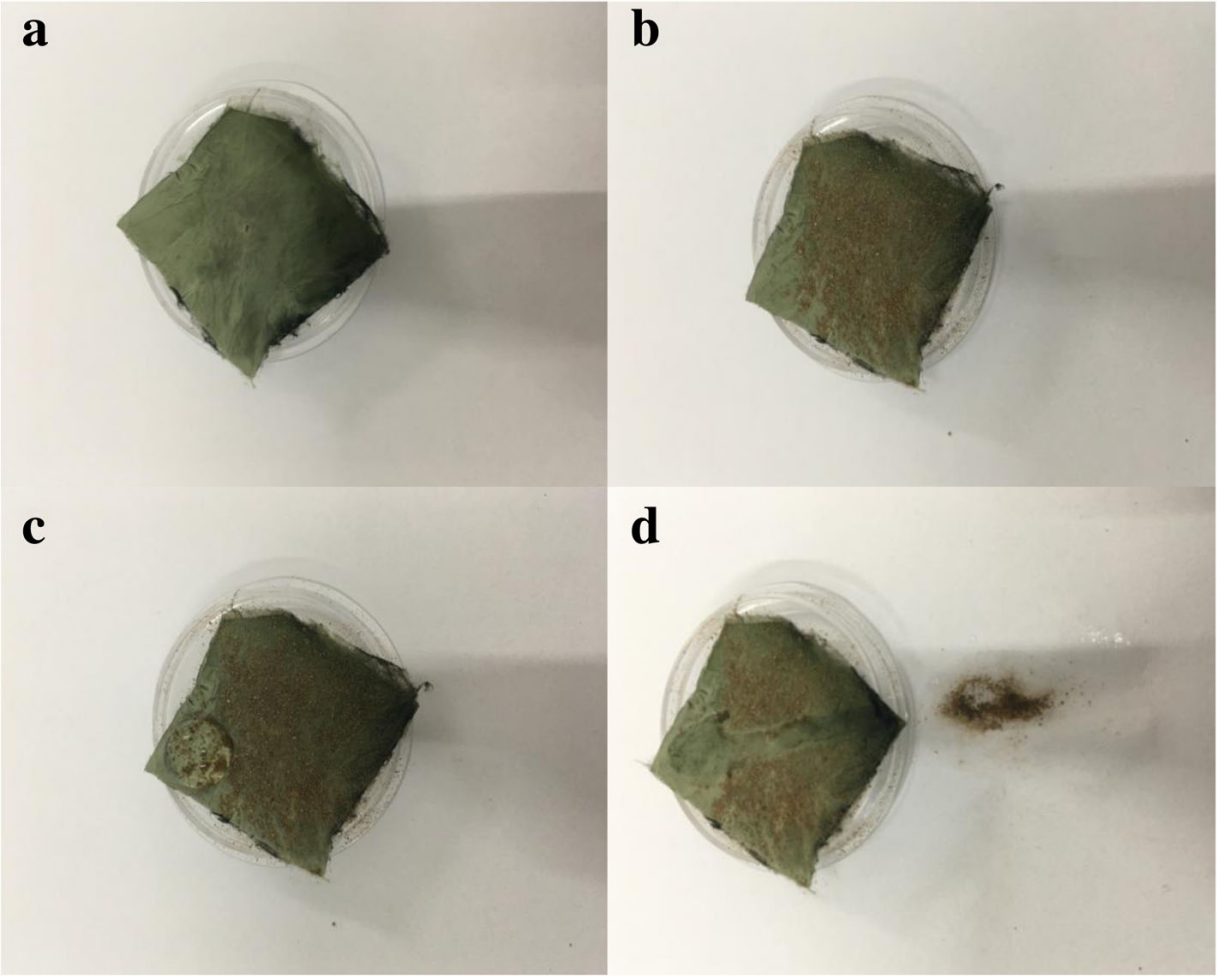
The optical photograph of a water drop rolling on the surface of Cu 1 to remove dust: **a** clean sample, **b** sample with dusty surface, **c** drop droplets on dust-covered samples, and **d** the droplets roll away the dust and expose the clean surface of the sample

Furthermore, due to the hydrophobicity of Cu 1 surface, water droplets can remain on the product surface. Therefore, the dust can be cleaned from the sample surface through rolling the water droplets on the surface to obtain self-cleaning effect. As displayed in Fig. 11a, before dropping water onto the surface of the sample, the sample presented a clean green surface. To show the self-cleaning effect, a layer of dust was scattered on the sample surface. Following it, a water droplet was dropped onto the surface of the sample. Slightly tilted the sample, the water droplet rolls on sample surface and brings dust down to present the original green surface. This means that in actual use, the prepared product can remove the attached dye or dust by sunlight or rain, thereby reducing maintenance costs.

## Conclusions

CuS nanoflowers were loaded TiO_2_/PVDF fibers through one-step hydrothermal method on electrospun TBOT/PVDF fibers. The method is very simple and convenient. In addition, the CuS/TiO_2_/PVDF fibers were prepared at lower temperature to ensure its flexibility. In the preparation process, the amount of Cu source determines the amount and crystalline quality of CuS supported on TiO_2_/PVDF fibers. When the amount of the Cu source reaches 1 mmol, the CuS supported on TiO_2_/PVDF fibers was a nanoflower-like structure formed by a hexagonal layer. The composite of CuS narrows the band gap energy of TiO_2_ and enhances the light harvest capability of TiO_2_. Besides, the composite of CuS increases the separation efficiency of the photogenerated electron-hole pairs of TiO_2_, correspondingly, improving the photocatalytic ability of TiO_2_ under visible light irradiation. The photocatalytic reaction rate of CuS/TiO_2_/PVDF fibers to RhB is 3 times higher than that of TiO_2_/PVDF fibers under visible light irradiation. In addition, after 5 times of recycle, the photocatalytic properties of CuS/TiO_2_/PVDF fibers did not decrease which mainly due to its flexibility and reusability. In addition, the as-prepared material has a very good self-cleaning effect on the dye and dust adhering to the surface, which can greatly reduce the maintenance cost of the material. It can be easily found that the as-prepared product not only has good photocatalytic activity but also has self-cleaning performance under visible light. The results presented in this paper provide a new perspective for exploring the application of novel flexible, recyclable, and self-cleaning photocatalytic materials for environmental sustainability.

## Additional file

Additional file 1: **Figure S1.** The optical photograph of the flexible CuS/TiO_2_/PVDF fibers. **Figure S2.** The spectrum of LED white light used in the experiment. **Figure S3.** XPS survey spectrum of the CuS/TiO_2_/PVDF fibers. **Figure S4.** The pseudo-first-order kinetics process of the as-prepared samples on photodegradation of RhB. **Figure S5.** Control experiments without and with radical scavengers for TiO_2_/PVDF (a) and CuS/TiO_2_/PVDF (b) fibers. (DOCX 1495 kb)

## Data Availability

The datasets generated during and/or analyzed during the current study are available from the corresponding authors on reasonable request.

## Abbreviations

CuS: Copper sulfide
DRS: Diffuse reflectance spectra
MB: Methyl blue
MO: Methyl orange
PL: Photoluminescence spectra
PVDF: Polyvinylidene fluoride
RhB: Rhodamine B
SEM: Scanning electron microscopy
TBOT: Tetrabutyl orthotitanate
TEM: Transmission electron microscopy
TiO_2_: Titanium dioxide
XPS: X-ray photoelectron spectroscopy
XRD: X-ray diffraction
