# Supplementary material for: One Step In Situ Loading of CuS Nanoflowers on Anatase TiO2/Polyvinylidene Fluoride Fibers and Their Enhanced Photocatalytic and Self-Cleaning Performance
Source: Nanoscale Res Lett. 2019 Jun 25;14:215. doi: 10.1186/s11671-019-3052-5 (PMC6592988; doi:10.1186/s11671-019-3052-5)
Supplement: Supplementary file 1 — Figure S1. The optical photograph of the flexible CuS/TiO2/PVDF fibers. Figure S2. The spectrum of LED white light used in the experiment. Figure S3. XPS survey spectrum of the CuS/TiO2/PVDF fibers. Figure S4. The pseudo-first-order kinetics process of the as-prepared samples on photodegradation of RhB. Figure S5. Control experiments without and with radical scavengers for TiO2/PVDF (a) and CuS/TiO2/PVDF (b) fibers. (DOCX 1495 kb) [file 11671_2019_3052_MOESM1_ESM.docx]

**One Step in Situ Loading of CuS Nanoflowers on Anatase TiO_2_/PVDF Fibers and Their Enhanced Photocatalytic and Self-cleaning Performance**

Zhi-Guang Zhang,^a),b)^ Hui Liu,^a)^ Yu-Qian Cui,^c)^ Min Dong,^c)^ Qing-Hao Li,^c)^ Xiao-Xiong Wang,^a)^ Seeram Ramakrishna,^a),d)^ and Yun-Ze Long ^a)*^

**Fig. S1** The optical photograph of the flexible CuS/TiO_2_/PVDF fibers.

**Fig. S2** The spectrum of LED white light used in the experiment.

**Fig. S3** XPS survey spectrum of the CuS/TiO_2_/PVDF fibers.

**Fig. S4** The pseudo-first-order kinetics process of the as-prepared samples on photodegradation of RhB.

**Fig. S5** Control experiments without and with radical scavengers for TiO_2_/PVDF (a) and CuS/TiO_2_/PVDF (b) fibers.
